# Effects of acute hypoxic exposure on multiple-object tracking ability of flight trainees

**DOI:** 10.3389/fpsyg.2026.1773817

**Published:** 2026-04-10

**Authors:** Zhi Xu, Nianqian Li, Xin Gao, Xiuyi Li, Wei Wu, Xiaolong Li, Yuchuan Luo, Yu Meng, Xi Chen, Xin Peng

**Affiliations:** 1 Civil Aviation Flight University of China, Guanghan, China; 2 Sichuan Provincial Engineering Research Center of Domestic Civil Aircraft Flight and Operation Support, Civil Aviation Flight University of China, Guanghan, China; 3 School of Aeronautics and Astronautics, Xihua University, Chengdu, China; 4 Engineering Research Center of Intelligent Air-Ground Integration Vehicle and Control, Xihua University, Ministry of Education, Chengdu, China

**Keywords:** acute hypoxia, civil aviation pilot, dynamic visual attention, flight safety, high-altitude flight, human factors, multiple-object tracking

## Abstract

**Introduction:**

Acute hypoxic exposure can threaten flight safety by impairing the cognitive function of civil aviation pilots. This study aimed to investigate the effects of acute moderate hypoxia on pilots’ dynamic visual attention using a multiple-object tracking (MOT) paradigm.

**Methods:**

Twenty-nine male flight trainees were recruited to perform the MOT task while exposed to a simulated hypoxic environment. Their reaction time and accuracy in each trial were recorded.

**Results:**

When tracking four target disks, participants’ accuracy was significantly lower under hypoxic conditions compared with normoxic conditions. Meanwhile, when tracking six targets, their reaction times significantly shortened.

**Discussion:**

The results indicate that hypoxia can lower the upper limit of multiple-object tracking capacity from four objects to three. Hypoxia appears to shift the speed-accuracy trade-off toward faster responses at the expense of accuracy. First-click response indicates that flight trainees with better tracking skills tended to display faster and more accurate first-click responses. The negative impact of hypoxia on participants varies between individuals and is more pronounced when tracking a larger number of objects. These findings provide a theoretical basis for improving the selection and hypoxic training of pilots flying at high altitude and for optimizing cockpit human-machine interaction interfaces.

## 1 Introduction

Safe flight operations critically depend on pilots’ cognitive functioning, particularly under physiologically demanding conditions. In high-altitude environments, reduced ambient oxygen availability can lead to acute hypoxia, which has been shown to impair cognitive and psychomotor performance (Post et al., 2023). Even moderate hypoxic exposure may therefore pose a potential threat to flight safety. Acute hypoxia has been associated with declines in higher-order cognitive functioning, psychomotor slowing, visual disturbances, and nonspecific psychological symptoms. Among these effects, attentional deficits as the most frequently self-reported cognitive complaint (Smith, 2008). Given that aviation is a highly attention-intensive domain, even subtle hypoxia-induced disruptions in attentional functioning may have important operational consequences.

During flight operations, pilots may be exposed to hypoxic conditions for various reasons. Operations on high-altitude routes often involve higher cruising altitudes, increasing the likelihood of reduced oxygen availability. Under abnormal circumstances, such as cabin depressurization, flight crews may consequently face severe hypobaric hypoxia. Several historical aviation events illustrate these risks. For example, the crash of Helios Airways Flight 522 resulted from unrecognized cabin depressurization, which ultimately led to crew incapacitation due to hypoxia (Air Accident Investigation and Aviation Safety Board [AAIASB], 2006). In another incident, the cockpit windshield rupture on Sichuan Airlines Flight 3 U8633 exposed the flight crew to extreme high-altitude environmental stressors, including reduced pressure and oxygen availability (Civil Aviation Administration of China [CAAC], 2020). These events highlight that acute hypoxic exposure remains a realistic and safety-relevant threat in civil aviation.

Experimentally, high-altitude hypoxia is commonly simulated using either hypobaric hypoxia (HH), produced in hypobaric chambers, or normobaric hypoxia (NH), induced by reducing inspired oxygen concentration while maintaining ambient pressure. HH more closely replicates real high-altitude atmospheric conditions and thus provides greater ecological validity. However, NH is more economical and easier to implement and is consequently widely used in laboratory studies (Gerhart et al., 2019; Liu et al., 2022; Zani et al., 2023). Although some physiological differences between HH and NH have been reported, converging evidence suggests that reduced oxygen availability itself is the primary driver of cognitive impairment. Barometric pressure appears to exerts relatively limited independent effects under controlled comparisons (Aebi et al., 2020; Shaw et al., 2021). Accordingly, the present study employed a NH protocol to isolate the cognitive consequences of acute oxygen deprivation.

Human factors remain the leading contributors to aviation accidents and incidents, and pilots play a central role in maintaining flight safety. Approximately 80% of operational information during flight is acquired through visual channels (Gao and Wang, 2024). A recent systematic review reported that several components of attention, including sustained and divided attention, are vulnerable to hypoxic exposure, particularly when blood oxygen saturation falls below 80% (Post et al., 2023). Furthermore, when cognitively demanding tasks involve visual search and substantial attentional requirements, higher-order cognitive processes such as inhibitory control and working memory appear particularly susceptible to hypoxia (Borden et al., 2024). In military aviation, dedicated hypoxia awareness training is routinely conducted, during which hypoxia is deliberately induced to help pilots recognize their individual symptoms (Neuhaus and Hinkelbein, 2014; Bustamante-Sánchez et al., 2019). In contrast, although civil aviation pilots may receive theoretical instruction on hypoxia, systematic experimental research and practical training specifically targeting the cognitive risks of hypoxia remain relatively limited.

During actual flight operations, pilots must scan multiple instruments while continuously monitoring dynamically changing flight parameters. The information conveyed by these displays evolves over time, placing sustained demands on visual attentional resources. Dynamic visual attention—the capacity to simultaneously monitor and update multiple time-varying sources of information—therefore represents a critical cognitive function underlying safe flight operations. Li et al. (2016) reported longer fixation durations when pilots tracked moving targets compared with stationary ones, suggesting that processing dynamic visual information imposes greater cognitive demands. Although previous studies have made progress in examining the effects of hypoxia on cognitive functioning, less attention has been paid to how hypoxic environments affect civil aviation pilots’ ability to monitor dynamically changing flight information. Consequently, it remains unclear how hypoxic exposure may influence pilots’ multiple-object tracking performance when monitoring dynamic information.

The multiple-object tracking (MOT) paradigm provides a well-established method for assessing dynamic visual attention (Pylyshyn, 2001; Dye and Bavelier, 2010). Participants continuously track several independently moving targets among identical distractors in a MOT task, approximating dynamic monitoring demands encountered in real-world environments. Good performance in MOT tasks depends on the coordinated engagement of several cognitive processes, including selective attention, sustained attention, attentional allocation, and spatial working memory (Meyerhoff et al., 2017). Marion et al. (2025) further suggests that executive functions, such as inhibitory control and cognitive flexibility, may also contribute to tracking performance. Because attentional resources are limited yet flexibly distributed across multiple objects, the MOT paradigm offers a quantifiable framework for examining how attentional resources are allocated under cognitively demanding conditions.

Performance in attentionally demanding tasks often reflects a trade-off between processing speed and response accuracy. Steinman et al. (2023) reported that hypoxia increased pilots’ reaction time while largely preserving response accuracy. Fitts’ law characterizes the trade-off between movement speed and accuracy in goal-directed motor behavior, demonstrating that movement time increases as target size decreases and target distance increases (Fitts, 1954). The diversity-enabled sweet spots (DESSs) theory has provided a neural control account of the speed-accuracy trade-off, attributing it to constraints arising from neural transmission delays and information capacity limits (Nakahira et al., 2019). According to DESSs, the brain adopts a hierarchical control architecture that integrates a fast but low-precision reflexive layer with a slower yet high-precision planning layer, so that the system achieves a balance between speed and accuracy (Nakahira et al., 2021).

The present study aimed to examine the effects of acute hypoxic exposure on dynamic visual attention in civil aviation pilots. Using a NH protocol, performance on a MOT task was compared under hypoxic and normoxic conditions. We hypothesized that acute moderate hypoxia would impair attentional resource allocation, resulting in reduced tracking accuracy and prolonged response time. By focusing on a dynamic and operationally relevant cognitive paradigm in a trained aviation population, the present study seeks to extend current hypoxia research beyond static task measures and provide empirical evidence to inform aviation safety research and cognitive performance modeling.

## 2 Materials and methods

### 2.1 Procedures

This study adopted a 2 (oxygen condition: normoxia or hypoxia) × 6 (number of tracked target disks: 1, 2, 3, 4, 5, or 6) within-participants experimental design. Prior to the experiments, participants completed 12 practice trials of the MOT tasks to ensure they were proficient at completing the task and were familiar with the hypoxic environment, thereby minimizing practice effect. The formal experiment required participants to visit the laboratory only once, thus controlling for irrelevant variables such as sleep quality. There was a 20-min rest period between the two tests under different conditions (normoxia and hypoxia), and the order of the experimental conditions was randomized and counterbalanced across participants.

### 2.2 Participants

An *a priori* power analysis was conducted using G*Power for a two-factor repeated-measures ANOVA (within-within design). Assuming a medium effect size (*f* = 0.25), an alpha level of 0.05, and a desired statistical power of 0.90, with 12 repeated measurements (2 oxygen conditions × 6 target loads), a correlation among repeated measures of 0.5, and a non-sphericity correction (*ε*) of 0.75, the minimum required sample size was estimated to be 19 participants. This study recruited male flight trainees in the return-to-school phase (those who had completed their private pilot license course, instrument rating course, and commercial pilot license course) who were under a quasi-military management system to participate in the experiment. The inclusion criteria were as follows: (1) normal or corrected-to-normal vision; (2) residence at an altitude below 2,000 m; and (3) no medication use or alcohol consumption within 24 h prior to testing. Thirty participants were recruited for the experiment. One participant withdrew due to hypersensitivity to hypoxia. Data from the remaining 29 participants were included in the final analysis. Their mean age was 22.9 ± 0.6 years, and their mean cumulative flight experience was 237.7 ± 6.8 h. The study was approved by the Institutional Review Board of the Civil Aviation Flight University of China (approval date: September 26, 2025).

### 2.3 Hypoxia

The world’s highest civilian airport, Daocheng Yading Airport, is located at an altitude of 4,411 meters; accordingly, this study simulated an altitude of 4,500 meters. Participants wore breathing masks connected to high-pressure gas cylinders via pipes and pressure regulators and inhaled an 11.8% oxygen-nitrogen mixture. After resting for 5 min, SpO_2_ levels decreased to 75–80%, indicating that the simulation altitude had reached 4,500 meters. Using the BIOPAC MP150 channel physiological recording and analysis system, blood oxygen saturation, heart rate, and respiratory rate indicators were monitored and recorded to verify the simulation.

### 2.4 Multiple-object tracking (MOT) task

**Figure 1:**
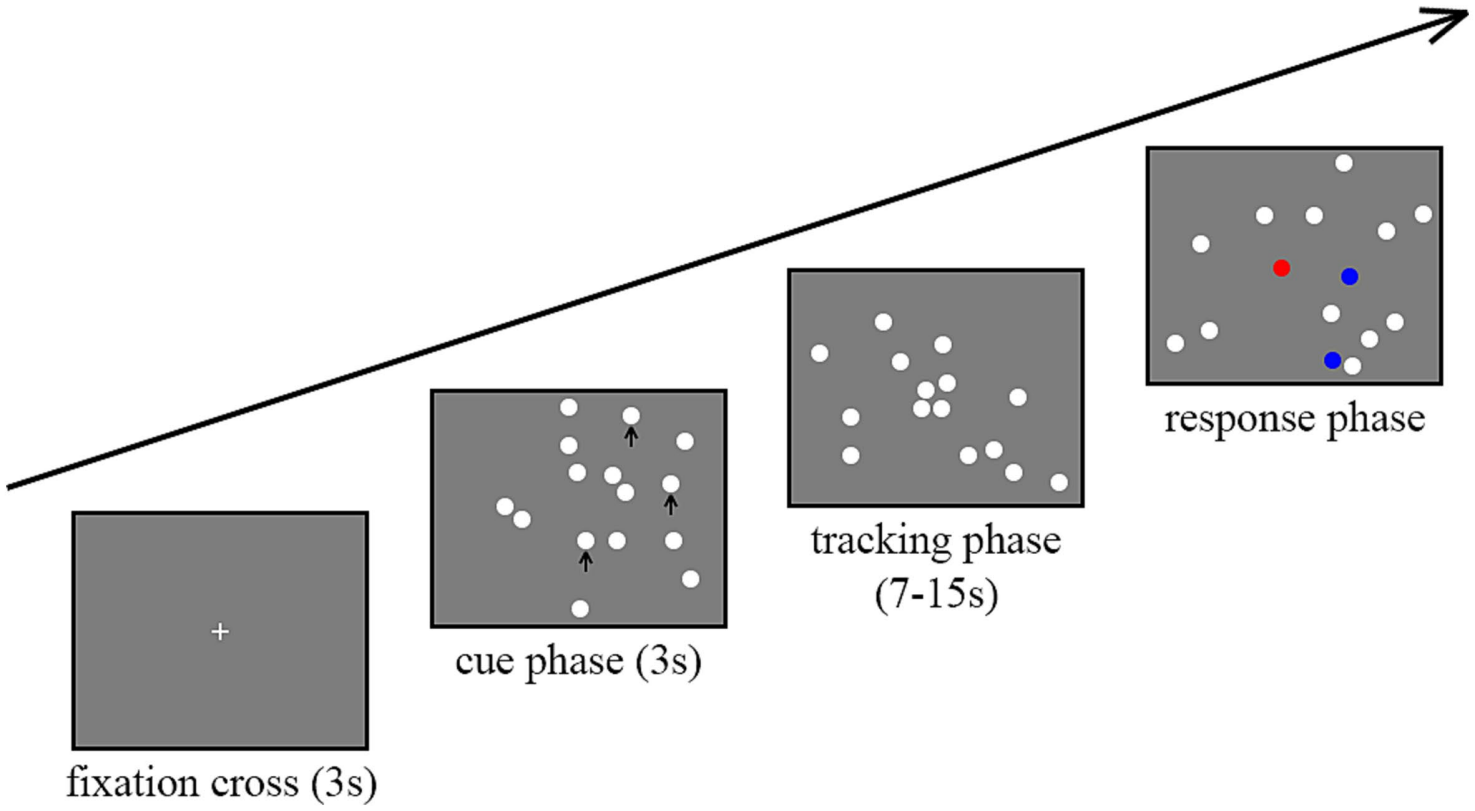
Experimental paradigm of the multiple-object tracking (MOT) task.

Multiple-object tracking (MOT) task consisted of three phases: the clue phase, the tracking phase, and the response phase (as shown in Figure 1). In the clue phase, at the start of the test, a white “+” fixation point appeared in the center of a gray window with a resolution of 1,280 × 1,024 pixels for 3 s, followed by the appearance of 15 white disks (diameter 10°). Among them, one to six target disks flashed for 3 s, indicating that they comprised the tracking targets. In the tracking phase, the target disks returned to the same state as the other disks and moved at a constant speed (3°/s) for 7–15 s before stopping. Each disk moved independently in a random straight line. The disks did not collide with each other but could overlap. In the response phase, participants used a mouse to click on the tracked targets. If a correct disk is tracked, it turned blue; otherwise, it turned red. When the number of clicks equaled the number of target disks, the next trial automatically began. Each target load (1–6 disk) appeared seven times in a random order for a total of 42 trials.

### 2.5 Indices

We recorded the accuracy and reaction time of each participant while completing the MOT test. Due to differences in the number of targets tracked, for ease of comparison and analysis, accuracy was categorized as follows: the mean accuracy within a single trial (abbreviated as mean accuracy $\bar{\mathrm{ACC}}$ ) and the accuracy of the first-click response in each trial (abbreviated as first-click accuracy $\mathrm{AC}C_{f}$ ). Additionally, reaction times were divided into the mean reaction time for each click within a single trial (abbreviated as mean reaction time $\bar{\mathrm{RT}}$ ), the total reaction time for a single trial (abbreviated as total reaction time $RT_{t}$ ), and the reaction time for the first-click in each trial (abbreviated as first-click reaction time $RT_{f}$ ).

### 2.6 Statistical analyses

Physiological signals acquired during the experiment were processed using AcqKnowledge (BIOPAC Systems, Inc.). The respiratory signal was filtered and smoothed to reduce noise, and respiratory cycles were identified to calculate respiratory rate (RR). The blood oxygen saturation (SpO₂) and heart rate (HR) signals were also smoothed to reduce transient fluctuations prior to statistical analysis.

Behavioral data were analyzed using SPSS. Mauchly’s test was conducted to examine the assumption of sphericity, and Greenhouse–Geisser corrections were applied when necessary. A repeated-measures analysis of variance (RMANOVA) was performed to compare task performance regarding accuracy and reaction time under normoxic and hypoxic conditions. Simple effect analyses were further conducted to prevent the overestimation or underestimation of the results that could occur upon relying on an interaction term (Kingsley et al., 2017). Here, ≥0.14 is considered a large effect size, ≥0.06 constitutes a medium effect size, and ≥0.01 denotes a small effect size (Fritz et al., 2012).

## 3 Results

### 3.1 Physiological responses

**Figure 2:**
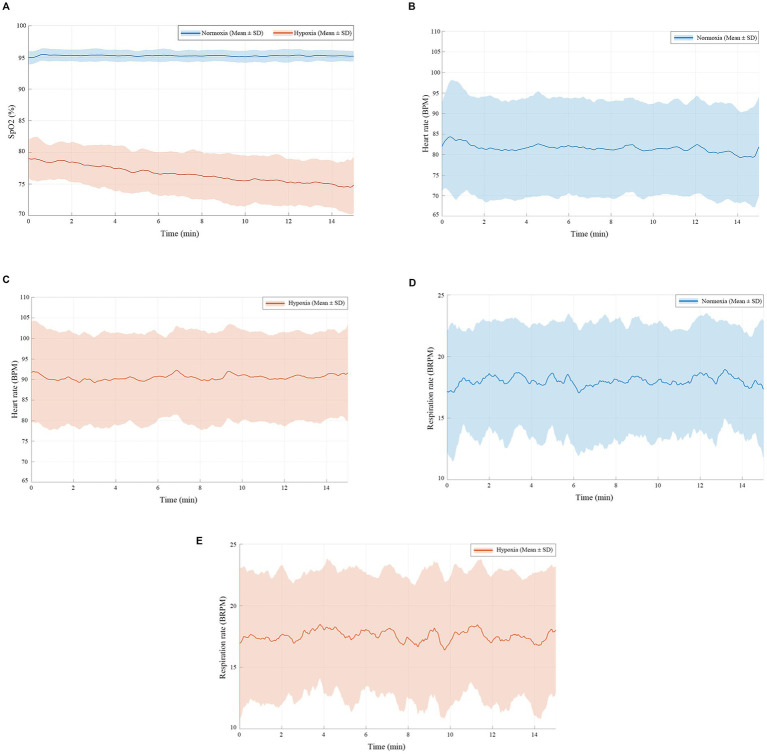
Physiological responses recorded during the task. **(A)** Blood oxygen saturation (SpO_2_) under normoxic and hypoxic conditions. **(B)** Heart rate (HR) under normoxia. **(C)** HR under hypoxia. **(D)** Respiratory rate (RR) under normoxia. **(E)** RR under hypoxia. Error bars represent standard deviations.

Physiological responses recorded during the task are shown in Figures 2A–E. The mean SpO₂ under normoxic conditions was 95.25 ± 0.73%, whereas under hypoxic conditions it decreased to 76.55 ± 3.17%, reaching the target simulated altitude. The mean HR was 81.56 ± 11.23 bpm under normoxia and 90.51 ± 10.52 bpm under hypoxia. The RR showed little difference between the two conditions, with values of 18.00 ± 2.29 brpm under normoxia and 17.53 ± 2.56 brpm under hypoxia.

### 3.2 Behavioral performance

#### 3.2.1 Mean accuracy ( $\bar{\mathrm{ACC}}$ )

**Figure 3:**
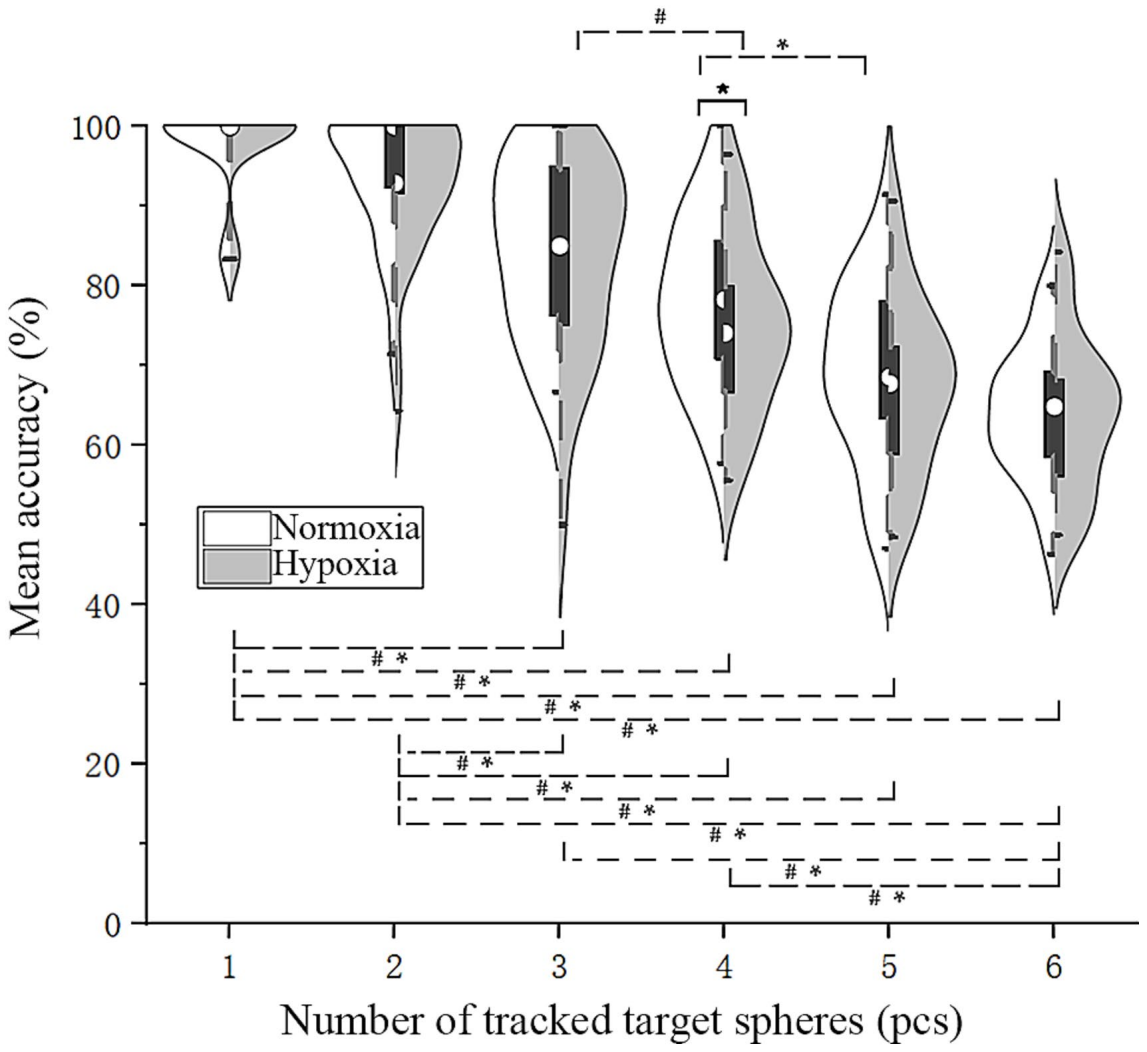
Violin plots of mean accuracy. Solid line (*): Simple effect, *p* < 0.05; dashed line (*#): pairwise comparisons under both oxygen conditions, *p* < 0.05; dashed line (*): pairwise comparisons under normoxic conditions, *p* < 0.05; dashed line (#): pairwise comparisons under hypoxic conditions, *p* < 0.05.

To determine whether hypoxic conditions decrease the number of targets that pilots are able to track, an RMANOVA was performed on $\bar{\mathrm{ACC}}$ under various oxygen conditions and different numbers of target disks, as shown in Figure 3. When there were four target disks, $\bar{\mathrm{ACC}}$ was significantly lower under hypoxic conditions. The main effect of oxygen conditions was not significant [*F*(1, 28) = 2.85, *p* = 0.103]. Mauchly’s test indicated that the assumption of sphericity was not violated for the number of tracked target disks [*χ*^2^(14) = 12.95, *p* = 0.53]. A repeated-measures ANOVA revealed a significant main effect of target number [*F*(5, 140) = 129.02, *p* < 0.001, $\eta_{p}^{2}$ = 0.82]. Similarly, Mauchly’s test confirmed that the sphericity assumption was met for the interaction term [*χ*^2^(14) = 18.78, *p* = 0.18]. The interaction between these two factors was not significant [*F*(5, 140) = 0.46, *p* = 0.81]. Further simple effect analysis revealed that under normoxic conditions, $\bar{\mathrm{ACC}}$ was significantly higher than under hypoxic conditions with four targets [*F*(1, 28) = 4.39, *p* = 0.045, $\eta_{p}^{2}$ = 0.14]. Bonferroni-adjusted pairwise comparisons revealed that, $\bar{\mathrm{ACC}}$ for one target was significantly higher than for three, four, five, and six targets (*p* < 0.001), and $\bar{\mathrm{ACC}}$ for two targets was significantly greater than for three, four, five, and six targets (*p* < 0.005). For three targets, it was significantly higher than for five and six targets (*p* < 0.001), and for four targets, it was significantly greater than for five targets (*p* < 0.005). Under normoxic conditions, $\bar{\mathrm{ACC}}$ for four targets was marginally significantly higher than for five (*p* = 0.006), while under hypoxic conditions, $\bar{\mathrm{ACC}}$ for four targets was significantly lower than for three (*p* = 0.002). These findings suggest that pilots may be unable to accurately track more than three objects under hypoxic conditions.

#### 3.2.2 First-click accuracy ( $\mathrm{AC}C_{f}$ )

**Figure 4:**
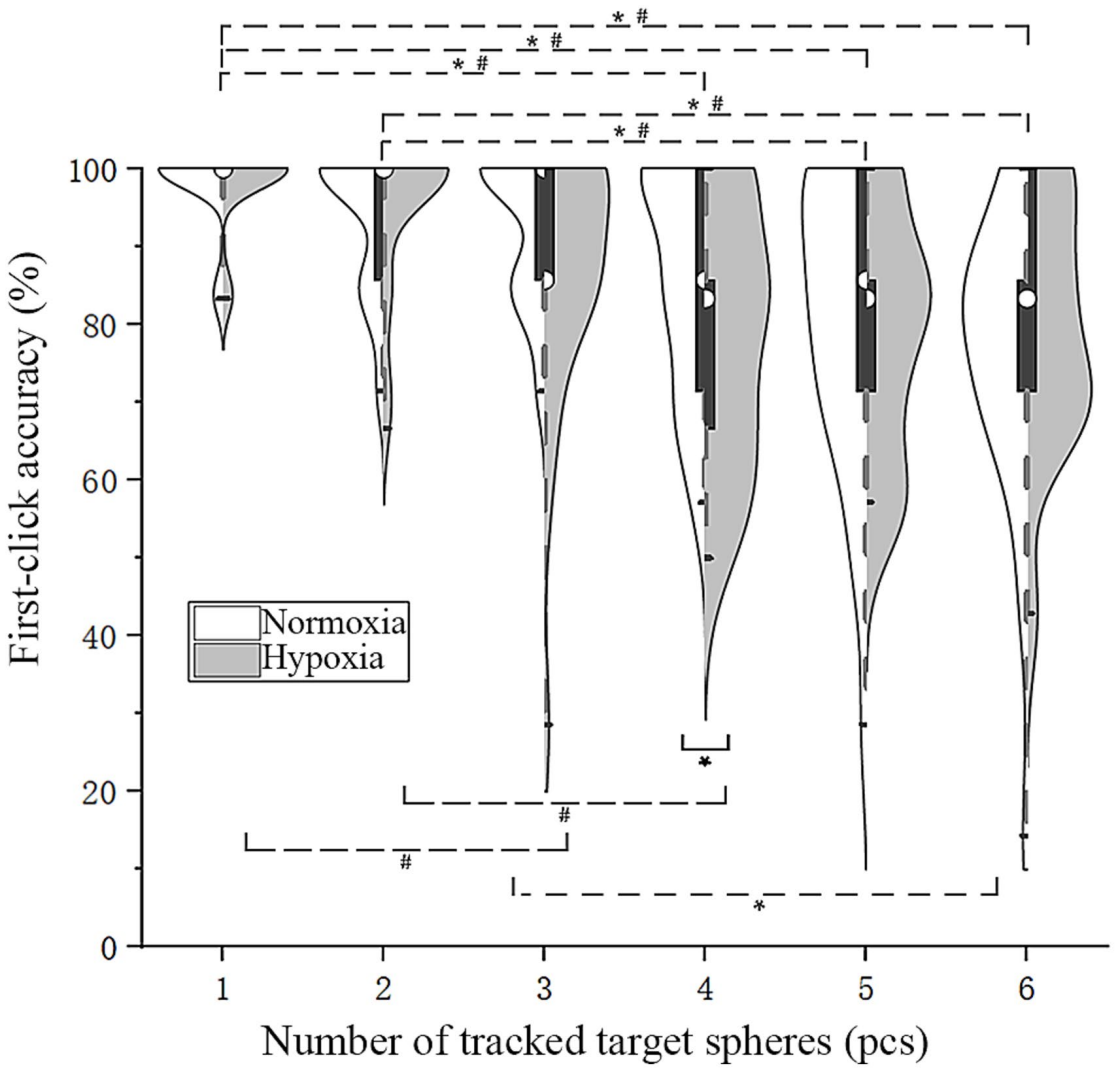
Violin plots of first-click accuracy. Solid line (*): simple effect, *p* < 0.05; dashed line (*#): Pairwise comparisons under both oxygen conditions, *p* < 0.05; dashed line (*): Pairwise comparisons under normoxic conditions, *p* < 0.05; dashed line (#): pairwise comparisons under hypoxic conditions, *p* < 0.05.

To specifically evaluate the first-click, which demonstrated longer reaction times, we isolated it for separate analysis. Following this, an RMANOVA was additionally carried out on $\mathrm{AC}C_{f}$ under different oxygen conditions and numbers of target disks (Figure 4). When participants tracked four target disks, $\mathrm{AC}C_{f}$ was significantly lower under hypoxic conditions. The main effect of oxygen conditions was not significant [*F*(1, 28) = 1.15, *p* = 0.29]. Mauchly’s test indicated that the assumption of sphericity was violated [*χ^2^*(14) = 29.54, *p* = 0.009]; therefore, Greenhouse–Geisser corrections were applied (*ε* = 0.74), whereas that of the number of tracked target disks was significant [*F*(3.71,103.97) = 21.91, *p* < 0.001, $\eta_{p}^{2}$ = 0.44]. Mauchly’s test indicated that the assumption of sphericity was not violated [*χ*^2^(14) = 19.26, *p* = 0.16]. The interaction between oxygen conditions and the number of tracked target disks was not significant [*F*(5, 140) = 1.81, *p* = 0.11]. Further simple effect analysis was conducted, indicating that under normoxic conditions, $\mathrm{AC}C_{f}$ was significantly higher than under hypoxic conditions when participants tracked four targets [*F*(1, 28) = 4.21, *p* = 0.050, $\eta_{p}^{2}$ = 0.13]. Bonferroni pairwise comparisons of $\mathrm{AC}C_{f}$ under the two oxygen conditions were performed, revealing that under both conditions, it was significantly higher for one target than for four, five, and six targets (*p* < 0.001), while for two targets, it was significantly higher than for five and six targets (*p* < 0.005). Under normoxic conditions, $\bar{\mathrm{ACC}}$ for three targets was significantly higher than for six targets (*p* = 0.001). In contrast, for one target under hypoxic conditions, $\bar{\mathrm{ACC}}$ was significantly higher than for three targets (*p* = 0.002), and $\bar{\mathrm{ACC}}$ for two targets was significantly higher than for four (*p* < 0.001). Consistent with the preceding findings, these results also indicate that pilots’ tracking capacity under hypoxic conditions is limited to a maximum of three targets. Additionally, these results suggest that the first-click response may reflect an individual’s tracking ability.

#### 3.2.3 Mean reaction time ( $\bar{\mathrm{RT}}$ )

**Figure 5:**
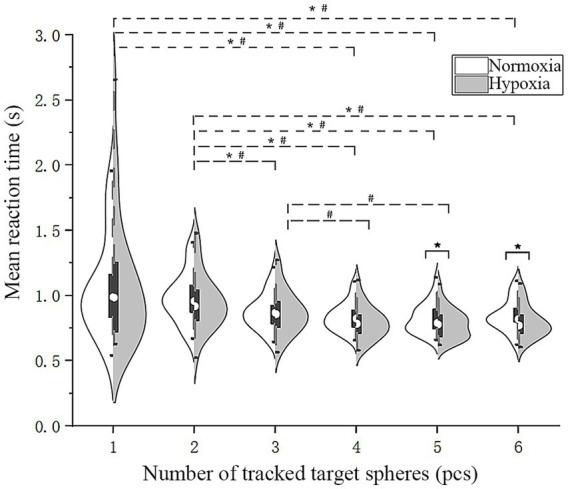
Violin plots of mean reaction time. Solid line (*): simple effect, *p* < 0.05; dashed line (*#): pairwise comparisons under both oxygen conditions, *p* < 0.05; dashed line (#): pairwise comparisons under hypoxic conditions, *p* < 0.05.

To investigate whether hypoxia exposure prolongs the mean task reaction time in pilots, as shown in Figure 5, we conducted an RMANOVA on $\bar{\mathrm{RT}}$ under various oxygen conditions and numbers of target disks. Upon using five and six target disks, $\bar{\mathrm{RT}}$ was significantly shorter under hypoxic conditions. The main effect of oxygen conditions was not significant [*F*(1, 28) = 1.52, *p* = 0.23]. Mauchly’s test indicated that the assumption of sphericity was violated [*χ*^2^(14) = 166.41, *p* < 0.001]; therefore, Greenhouse–Geisser corrections were applied (*ε* = 0.28), whereas that of the number of tracked target disks was significant [*F*(1.40, 39.29) = 14.13, *p* < 0.001, $\eta_{p}^{2}$ = 0.34]. Mauchly’s test likewise indicated a violation of the sphericity assumption [*χ*^2^(14) = 62.91, *p* < 0.001]; accordingly, Greenhouse–Geisser corrections were applied (*ε* = 0.46). The interaction between oxygen conditions and the number of tracked target disks was not significant [*F*(2.28, 63.87) = 0.77, *p* = 0.49]. We conducted a further simple effect analysis, which demonstrated that $\bar{\mathrm{RT}}$ under hypoxic conditions was significantly shorter than under normoxic conditions with five targets [*F*(1, 28) = 5.09, *p* = 0.032, $\eta_{p}^{2}$ = 0.15] and six targets [*F*(1, 28) = 5.53, *p* = 0.026, $\eta_{p}^{2}$ = 0.17]. Bonferroni pairwise comparisons of $\bar{\mathrm{RT}}$ under the two oxygen conditions were performed, revealing that for one target, it was significantly longer than four, five, and six targets (*p* < 0.005), and for two targets, it was significantly longer than three, four, five, and six (*p* < 0.005). Additionally, under hypoxic conditions, $\bar{\mathrm{RT}}$ for three targets was significantly greater than for four and five (*p* < 0.05). Unexpectedly, these findings indicate that participants’ reaction times actually shortened under hypoxic conditions.

#### 3.2.4 Total reaction time ( $RT_{t}$ )

**Figure 6:**
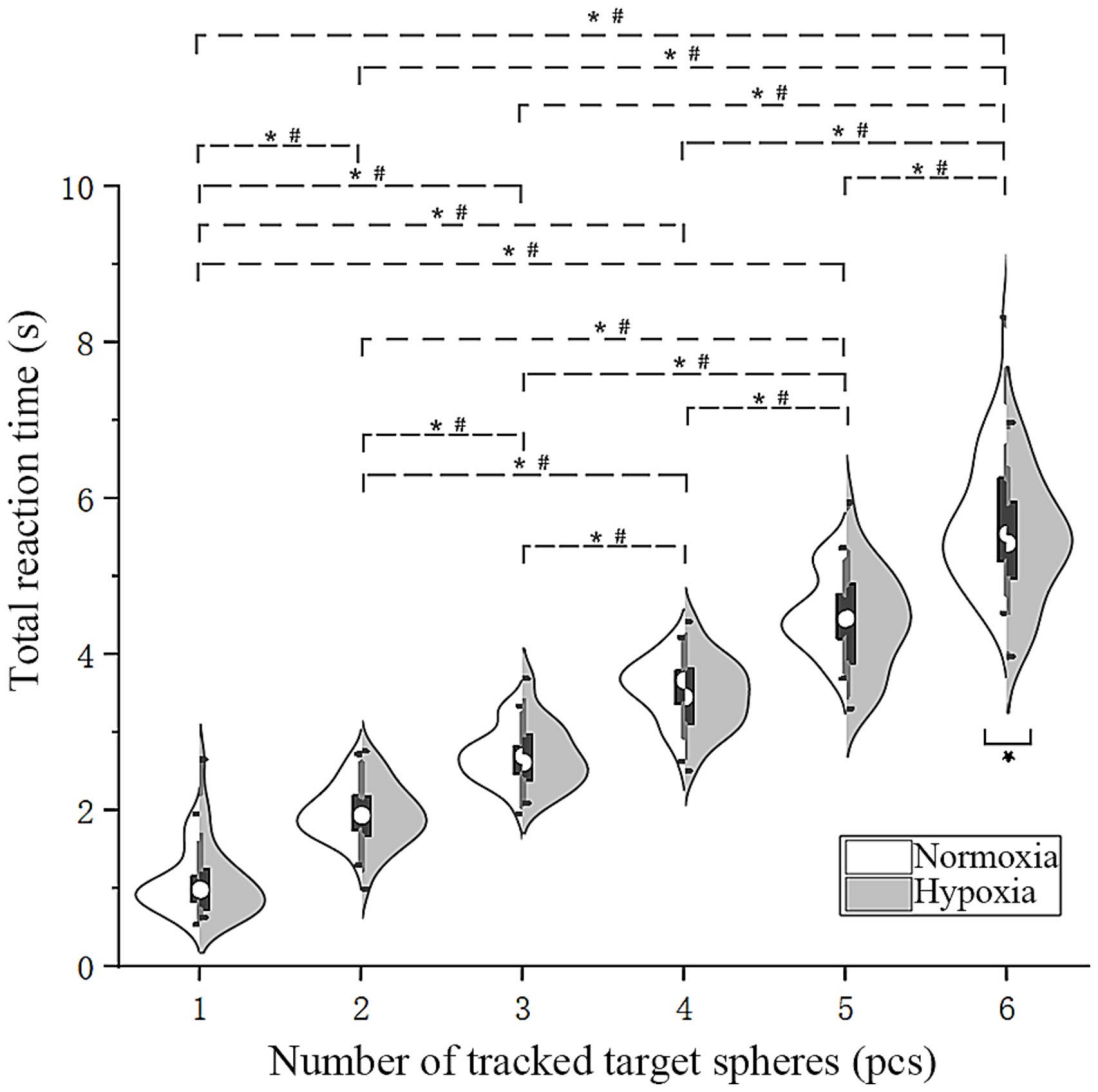
Violin plots of total reaction time. Solid line (*): simple effect, *p* < 0.05; dashed line (*#): pairwise comparisons under both oxygen conditions, *p* < 0.05.

To investigate the effects of hypoxia exposure on the total task reaction time of pilots, an RMANOVA was also performed on $RT_{t}$ under varied oxygen conditions and numbers of target disks (Figure 6). With six target disks, $RT_{t}$ was significantly shorter under hypoxic conditions. The main effect of oxygen conditions was not significant [*F*(1, 28) = 2.55, *p* = 0.12]. Mauchly’s test was significant, indicating a violation of the sphericity assumption [*χ^2^*(14) = 74.91, *p* < 0.001]; therefore, Greenhouse–Geisser corrections were applied (*ε* = 0.46). The main effect of the number of tracked target disks was significant [*F*(2.28, 63.74) = 765.60, *p* < 0.001, $\eta_{p}^{2}$ = 0.97]. Furthermore, Mauchly’s test indicated that the assumption of sphericity was violated [*χ*^2^(14) = 38.78, *p* < 0.001]; therefore, Greenhouse–Geisser corrections were applied (*ε* = 0.62). The interaction between oxygen conditions and the number of tracked target disks was not significant [*F*(3.09, 86.40) = 2.19, *p* = 0.93]. A further simple effect analysis showed that under hypoxic conditions, $RT_{t}$ was significantly shorter than under normoxic conditions when tracking six targets [*F*(1, 28) = 4.36, *p* = 0.046, $\eta_{p}^{2}$ = 0.14]. Bonferroni pairwise comparisons of $RT_{t}$ under different oxygen conditions revealed significant different oxygen conditions revealed significant differences for various numbers of tracked targets (*p* < 0.001). Consistent with the aforementioned findings, these results indicate that pilots’ reaction time actually shortened under hypoxic conditions.

#### 3.2.5 First-click reaction time ( $RT_{f}$ )

**Figure 7:**
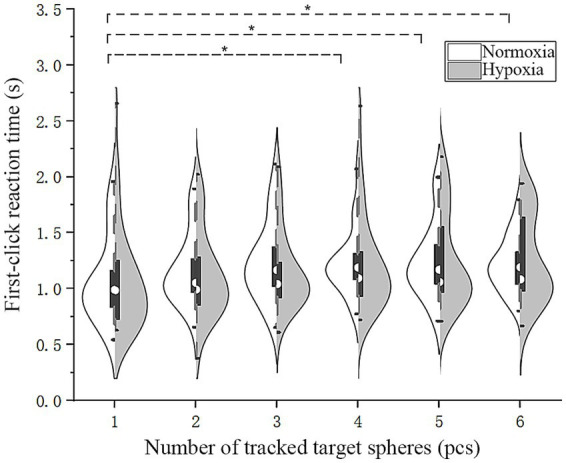
Violin plots of first-click reaction time. Dashed line (*): pairwise comparisons under normoxic conditions, *p* < 0.05.

To explore the effect of hypoxia exposure on pilots’ first-click response time, we additionally carried out an RMANOVA on $RT_{f}$ with various oxygen conditions and different numbers of target disks (Figure 7). The main effect of oxygen conditions was not significant [*F*(1, 28) = 0.04, *p* = 0.85]. Mauchly’s test indicated that the assumption of sphericity was violated [*χ*^2^(14) = 36.51, *p* = 0.001]; therefore, Greenhouse–Geisser corrections were applied (*ε* = 0.62). The main effect of the number of tracked target disks was significant [*F*(3.09, 86.55) = 4.78, p = 0.004, $\eta_{p}^{2}$ = 0.15]. Mauchly’s test indicated that the assumption of sphericity was not violated [*χ^2^*(14) = 21.71, *p* = 0.09]. The interaction between oxygen conditions and the number of tracked target disks was not significant [*F*(5, 140) = 1.01, *p* = 0.41]. Upon performing Bonferroni pairwise comparisons of $RT_{f}$ , it was revealed that under normoxic conditions, $RT_{f}$ for one target was significantly shorter than for four, five, and six targets (*p* < 0.05), while there was no significant difference in the number of targets tracked under hypoxic conditions. The results revealed that the first-click response time was shortest when tracking one target under normoxic conditions.

## 4 Discussion

This study conducted an MOT test to investigate the effects of acute hypoxia exposure on the multiple-object tracking ability of flight trainees, using a breathing mask to induce normobaric hypoxia and simulate conditions corresponding to an altitude of 4,500 meters. By analyzing the accuracy and reaction time indicators for tracking one to six target disks, we found that exposure to moderate hypoxia at atmospheric pressure may induce the following effects: (1) it may adversely affect flight trainees’ ability to track multiple objects, possibly suggesting a reduction in attentional resources and a lower upper limit of tracking capacity from four to three targets; (2) participants’ decision-making ability may be impaired, influencing the speed-accuracy trade-off and resulting in faster reaction times under hypoxic conditions but lower accuracy; (3) within each trial, the first-click response may reflect an individual’s multiple-object tracking ability, with more proficient trackers capable of faster and more accurate responses; and (4) the negative impact on multiple-object tracking performance varies across individuals and tends to be stronger in more demanding tasks.

In our study, acute hypoxia exposure impaired the ability of flight trainees to track multiple objects, possibly reducing their attentional resources and suggesting a reduction in tracking capacity from four to three. Previous studies report a limit of tracking four targets in MOT tests (Cowan, 2001; Alzahabi and Cain, 2021). In this study, there were no significant differences in $\bar{\mathrm{ACC}}$ (Figure 3) and $\mathrm{AC}C_{f}$ (Figure 4) between five and six tracked targets across both oxygen conditions, possibly due to a floor effect resulting from the task’s high level of difficulty; this supports the notion that four targets are the upper limit of tracking ability. Under hypoxic conditions and when tracking four targets, $\bar{\mathrm{ACC}}$ (Figure 3) and $\mathrm{AC}C_{f}$ (Figure 4) were significantly reduced, indicating that tracking ability may be more affected by hypoxia as it approaches its limits. As shown in Figure 3, under normoxic conditions, $\bar{\mathrm{ACC}}$ was significantly higher when tracking four targets than when tracking five, but not significantly different from three targets. In contrast, for four targets under hypoxic conditions, $\bar{\mathrm{ACC}}$ (Figure 3) was significantly lower than for three targets and showed no significant difference from five targets. This indicates that acute hypoxic exposure may reduce participants’ attentional resources, thereby lowering their effective tracking limit from four to three targets. It is recommended that pilots achieve an accuracy of approximately 85% when tracking three targets and 80% when tracking four targets under hypoxic conditions equivalent to 4,500 m altitude.

The relationship between reaction time and accuracy under hypoxia cannot be reduced to a simple compensatory trade-off. Instead, the present findings suggest a shift toward faster but less accurate responding, possibly reflecting a relative lowering of decision thresholds. Specifically, under hypoxic conditions and increasing task load, $\bar{\mathrm{ACC}}$ (Figure 3) and $\mathrm{AC}C_{f}$ (Figure 4) decreased when tracking four targets, whereas $\bar{\mathrm{RT}}$ (Figure 5) decreased when tracking five and six targets, and $RT_{t}$ (Figure 6) decreased when tracking six targets. This pattern suggests constrained control rather than strategic optimization. Acute hypoxia has been reported to induce subtle subjective states, such as heightened excitement, reduced inhibition, and transient well-being (Shaw et al., 2021). Such states may promote speed-oriented responding without awareness of declining accuracy. Importantly, no instructions emphasizing speed or accuracy were provided in the present experiment, indicating that the observed shift was unlikely to be instruction-driven and may instead reflect physiological influences. The DESSs framework proposes that hierarchical neural architectures enable optimization of the speed-accuracy balance under normoxic conditions (Nakahira et al., 2021). Hypoxia may preferentially impair higher-order executive processes, thereby shifting processing toward faster but less controlled response modes. In contrast to Steinman et al. (2023), who reported prolonged reaction times under hypoxia consistent with compensatory strategy adjustment, the present results revealed shortened reaction times accompanied by decreased accuracy. Although the behavioral patterns differ, both sets of findings converge in suggesting that hypoxia alters attentional control mechanisms. The higher cognitive demands of the MOT task used in the present study may have limited participants’ capacity to strategically preserve accuracy.

The first-click responses may reflect the efficiency of multiple-object tracking, with individuals showing stronger tracking performance tending to produce faster and more accurate first-click responses. In the present experiment, reaction time for the first click was significantly longer than for subsequent clicks. This likely reflects the absence of motor preparation during the tracking phase. Consistent with Fitts’s law, a larger movement amplitude would be expected to produce longer response times. In addition to motor factors, the first click may involve a cognitive transition from global tracking to selecting a specific target. Notably, first-click accuracy was not perfect, suggesting that performance at this stage may partially reflect the overall quality of tracking during the preceding phase. When comparing $\mathrm{AC}C_{f}$ (Figure 4) and $RT_{f}$ (Figure 7), tracking a single target under normoxic conditions resulted in higher accuracy and shorter reaction times than tracking four, five, or six targets. The MOT task requires the maintenance and processing of spatial information in visual working memory (Zhang et al., 2010), and the first click involves the retrieval of a target’s spatial representation. Targets that were successfully maintained during tracking can be retrieved more efficiently, resulting in both faster and more accurate responses. In contrast, when tracking fails and spatial representations become uncertain, extended retrieval time does not necessarily improve accuracy. Therefore, the first-click response may partially reflect the overall quality of tracking during the preceding phase.

The adverse effects of hypoxia on multiple-object tracking ability varied between individuals, becoming more pronounced in tasks that were more challenging. The distribution of $RT_{f}$ values appeared more spread out under hypoxia, especially in the six-target condition (Figure 7). Under high attentional load, hypoxic stress may amplify pre-existing individual differences in cognitive resource capacity. Moreover, during the experiment, one participant elected to discontinue the experiment prematurely due to his heightened sensitivity to hypoxia. Nation et al. (2017) also found significant individual differences in the effects of exposure to high altitude on cognition, with some crew members showing no significant changes in cognition, while others showed a significant decline. Li et al. (2025) found that pilots with better gas exchange efficiency and lung function could endure acute high-altitude hypoxia for longer. Leinonen et al. (2024) found that after undergoing normobaric hypoxic training, pilots’ ability to recognize hypoxic symptoms improved; however, 23% of the participants were slow to recognize symptoms and did not show improvement even after repeated training.

The present study has several limitations that should be acknowledged. The scarcity of flight trainees resulted in a relatively small sample size. Given that the current aviation workforce is predominantly composed of male pilots, only male participants were included. Future studies including female pilots would help improve the generalizability of the findings. The simulated hypoxic environment was created by diluting oxygen with nitrogen under normobaric conditions. Future research could further investigate the effects of hypobaric hypoxia using hypobaric chambers. Only SpO₂, was monitored in the present study. However, central oxygenation may play an important role in cognitive performance during hypoxic exposure. Incorporating measures of cerebral oxygen saturation in future research may therefore provide a more comprehensive physiological assessment. Visual cognition was assessed primarily through attention-related performance in the MOT task. Integrating eye-tracking measures in future studies may provide additional insight into visual attention allocation under hypoxic conditions. This study manipulated only the number of targets in the MOT task, while other factors such as target speed were fixed. As a result, the task lacked key contextual elements of real flight, limiting the applicability of the results to in-cockpit performance. Future research could vary target movement speed and patterns to more closely emulate the dynamic demands of real flight. The present findings suggest that hypoxia may influence the speed-accuracy trade-off. Future studies could manipulate factors such as task instructions or time pressure to further examine how hypoxia affects decision strategies related to speed and accuracy.

## 5 Conclusion

This study examined the performance of civil aviation flight trainees on a MOT task under NH simulating an altitude of 4,500 m. The results indicate that acute moderate hypoxia impairs the allocation of attentional resources. Contrary to our initial hypothesis, hypoxic exposure reduced tracking accuracy while shortening response times, indicating a compromised speed-accuracy trade-off. These findings underscore the importance of hypoxia recognition and tolerance training for pilots in order to maintain an appropriate balanced between speed and accuracy and to support rapid yet effective responses during hypoxia-related emergencies. In addition, variability in performance across individuals was observed under hypoxic conditions, suggesting that individual sensitivity to hypoxia may be a relevant factor to consider when selecting pilots for high-altitude route operations. Given that hypoxia reduced the effective capacity of multiple-object tracking from four targets to three, human-machine interface design, including both hardware displays and software interfaces, should limit the number of tasks that need to be monitored simultaneously to no more than three.

## Data Availability

The datasets presented in this article are not readily available because of privacy and ethical restrictions. The datasets presented in this study are available on request from the corresponding author. Requests to access the datasets should be directed to Zhi Xu, hughesxu@aliyun.com.

## References

[ref1] Aebi M. R. Bourdillon N. Noser P. Millet G. P. Bron D. (2020). Cognitive impairment during combined normobaric vs. hypobaric and normoxic vs. hypoxic acute exposure. Aerospace Med Hum Perfor 91, 845–851. doi: 10.3357/amhp.5616.2020, 33334404

[ref2] Air Accident Investigation and Aviation Safety Board [AAIASB] (2006) Aircraft Accident Report: Helios Airways Flight HCY522 Boeing 737-31S at Grammatiko, Hellas on 14 August 2005

[ref3] Alzahabi R. Cain M. S. (2021). Ensemble perception during multiple-object tracking. Atten. Percept. Psychophys. 83, 1263–1274. doi: 10.3758/s13414-020-02219-4, 33409901 PMC8049938

[ref4] Borden C. K. McHail D. G. Blacker K. J. (2024). The time course of hypoxia effects using an aviation survival trainer. Front. Cogn. 3:1375919. doi: 10.3389/fcogn.2024.1375919 PMC13281091 42339413

[ref5] Bustamante-Sánchez Á. Delgado-Terán M. Clemente-Suárez V. J. (2019). Psychophysiological response of different aircrew in normobaric hypoxia training. Ergonomics 62, 277–285. doi: 10.1080/00140139.2018.1510541, 30101685

[ref6] Civil Aviation Administration of China [CAAC] (2020). Investigation Report on the Sichuan Airlines Flight 3U8633 (A319-100 / B-6419) Windscreen Shattered and Detached En Route from Chongqing to Lhasa on May 14, 2018

[ref7] Cowan N. (2001). The magical number 4 in short-term memory: a reconsideration of mental storage capacity. Behav. Brain Sci. 24, 87–114. doi: 10.1017/S0140525X01003922, 11515286

[ref8] Dye M. W. G. Bavelier D. (2010). Differential development of visual attention skills in school-age children. Vis. Res. 50, 452–459. doi: 10.1016/j.visres.2009.10.010, 19836409 PMC2824025

[ref9] Fitts P. M. (1954). The information capacity of the human motor system in controlling the amplitude of movement. J. Exp. Psychol. 47, 381–391. doi: 10.1037/h0055392, 13174710

[ref10] Fritz C. O. Morris P. E. Richler J. J. (2012). Effect size estimates: current use, calculations, and interpretation. J. Exp. Psychol. Gen. 141, 2–18. doi: 10.1037/a0024338., 21823805

[ref11] Gao S. Wang L. (2024). How flight experience impacts pilots’ decision-making and visual scanning pattern in low-visibility approaches: preliminary evidence from eye tracking. Ergonomics 67, 1284–1300. doi: 10.1080/00140139.2023.2298992., 38254322

[ref12] Gerhart H. D. Seo Y. Kim J. H. Followay B. Vaughan J. Quinn T. . (2019). Investigating effects of cold water hand immersion on selective attention in Normobaric hypoxia. Int. J. Environ. Res. Public Health 16. doi: 10.3390/ijerph16162859, 31405091 PMC6720274

[ref14] Kingsley A. F. Noordewier T. G. Vanden Bergh R. G. (2017). Overstating and understating interaction results in international business research. J. World Bus. 52, 286–295. doi: 10.1016/j.jwb.2016.12.010.

[ref15] Leinonen A. M. Varis N. O. Kokki H. J. Leino T. K. (2024). Normobaric hypoxia symptom recognition in three training sessions. Aerosp. Med. Hum. Perform. 95, 758–764. doi: 10.3357/amhp.6380.2024., 39431701

[ref16] Li B. Xu Y. Wang D. Wei B. Zhu H. Wu M. . (2025). High-altitude acute hypoxia endurance and comprehensive lung function in pilots. Aerospace Med Hum Perfor 96, 191–197. doi: 10.3357/amhp.6551.2025, 40029347

[ref17] Li W. C. Yu C. S. Braithwaite G. Greaves M. (2016). Pilots' attention distributions between chasing a moving target and a stationary target. Aerosp. Med. Hum. Perform. 87, 989–995. doi: 10.3357/amhp.4617.2016, 28323583

[ref18] Liu J. Li S. Liu M. Xu X. Zhang Y. Cheng J. . (2022). Impaired brain networks functional connectivity after acute mild hypoxia. Medicine 101:e30485. doi: 10.1097/md.0000000000030485, 36197178 PMC9509199

[ref19] Marion P. Maxime A. Pierre-Yves O. Hélène S. (2025). Broadening the lens: a review of multi-object tracking task and its use in cognitive training. Acta Psychol. 258:105271. doi: 10.1016/j.actpsy.2025.105271 40652893

[ref20] Meyerhoff H. S. Papenmeier F. Huff M. (2017). Studying visual attention using the multiple object tracking paradigm: a tutorial review. Atten. Percept. Psychophys. 79, 1255–1274. doi: 10.3758/s13414-017-1338-1, 28584953

[ref21] Nakahira Y. Liu Q. Sejnowski T. J. Doyle J. C. (2019). Fitts' law for speed-accuracy trade-off describes a diversity-enabled sweet spot in sensorimotor control. [Epubh ahead of preprint]. doi: 10.48550/arXiv.1906.00905. PMC8179159 34050009

[ref22] Nakahira Y. Liu Q. Sejnowski T. J. Doyle J. C. (2021). Diversity-enabled sweet spots in layered architectures and speed–accuracy trade-offs in sensorimotor control. Proc. Natl. Acad. Sci. 118:e1916367118. doi: 10.1073/pnas.1916367118, 34050009 PMC8179159

[ref23] Nation D. A. Bondi M. W. Gayles E. Delis D. C. (2017). Mechanisms of memory dysfunction during high altitude hypoxia training in military aircrew. J. Int. Neuropsychol. Soc. 23, 1–10. doi: 10.1017/s1355617716000965, 27923417 PMC5472446

[ref24] Neuhaus C. Hinkelbein J. (2014). Cognitive responses to hypobaric hypoxia: implications for aviation training. Psychol. Res. Behav. Manag. 7, 297–302. doi: 10.2147/PRBM.S51844, 25419162 PMC4234165

[ref25] Post T. E. Heijn L. G. Jordan J. van Gerven J. M. A. (2023). Sensitivity of cognitive function tests to acute hypoxia in healthy subjects: a systematic literature review. Front. Physiol. 14:1244279. doi: 10.3389/fphys.2023.1244279 37885803 PMC10598721

[ref26] Pylyshyn Z. W. (2001). Visual indexes, preconceptual objects, and situated vision. Cognition 80, 127–158. doi: 10.1016/S0010-0277(00)00156-6, 11245842

[ref27] Shaw D. M. Cabre G. Gant N. (2021). Hypoxic hypoxia and brain function in military aviation: basic physiology and applied perspectives. Front. Physiol. 12. doi: 10.3389/fphys.2021.665821, 34093227 PMC8171399

[ref28] Smith A. M. (2008). Hypoxia symptoms in military aircrew: long-term recall vs. acute experience in training. Aviat. Space Environ. Med. 79, 54–57. doi: 10.3357/asem.2013.2008, 18225780

[ref29] Steinman Y. Groen E. Frings-Dresen M. H. W. (2023). Hypoxia impairs reaction time but not response accuracy in a visual choice reaction task. Appl. Ergon. 113:7. doi: 10.1016/j.apergo.2023.104079 37413961

[ref30] Zani A. Crotti N. Marzorati M. Senerchia A. Proverbio A. M. (2023). Acute hypoxia alters visuospatial attention orienting: an electrical neuroimaging study. Sci. Rep. 13:22746. doi: 10.1038/s41598-023-49431-4, 38123610 PMC10733389

[ref001] Zhang H. Xuan Y. Fu X. Pylyshyn Z. W. (2010). Do objects in working memory compete with objects in perception?. Vis. cogn. 18, 617–640. doi: 10.1080/13506280903211142, 34050009

